# The pathogenesis and therapeutic strategies of heat stroke-induced liver injury

**DOI:** 10.1186/s13054-022-04273-w

**Published:** 2022-12-17

**Authors:** Fuquan Wang, Yan Zhang, Jianhua Li, Haifa Xia, Dingyu Zhang, Shanglong Yao

**Affiliations:** 1 Department of Anesthesiology, Union Hospital, Tongji Medical College, Huazhong University of Science and Technology, Wuhan, 430022 China; 2 Department of Anesthesiology, Institute of Anesthesia and Critical Care Medicine, Union Hospital, Tongji Medical College, Huazhong University of Science and Technology, No. 1277, Jiefang Avenue, Wuhan, 430022 China; 3 Chongqing university Jiangjin hospital, Chongqing, China; 4 Wuhan Jinyintan Hospital, Wuhan, 430023 China

**Keywords:** Heat stroke, Liver injury, Pathogenesis, Therapeutic strategy

## Abstract

Heat stroke (HS) is a life-threatening systemic disease characterized by an elevated core body temperature of more than 40 ℃ and subsequent multiple organ dysfunction syndrome. With the growing frequency of global heatwaves, the incidence rate of HS has increased significantly, which has caused a huge burden on people's lives and health. Liver injury is a well-documented complication of HS and usually constitutes the direct cause of patient death. In recent years, a lot of research has been carried out on the pathogenesis and treatment strategies of HS-induced liver injury. In this review, we summarized the important pathogenesis of HS-induced liver injury that has been confirmed so far. In addition to the comprehensive effect of systemic factors such as heat cytotoxicity, coagulopathy, and systemic inflammatory response syndrome, excessive hepatocyte cell pyroptosis, dysfunction of Kupffer cells, abnormal expression of heat shock protein expression, and other factors are also involved in the pathogenesis of HS-induced liver injury. Furthermore, we have also established the current therapeutic strategies for HS-induced liver injury. Our study is of great significance in promoting the understanding of the pathogenesis and treatment of HS-induced liver injury.

## Introduction

Heatstroke (HS) was a severe heat illness characterized by an elevated core body temperature exceeding 40 °C and often accompanied by central nervous system dysfunction and multiple organ injury such as liver, intestine, and lung [1, 2]. With the continuous warming of the global climate and the frequent occurrence of extreme weather in summer, the incidence and mortality of HS have shown an obvious upward trend globally [3]. Accordingly, HS has attracted more and more researchers' attention in recent years. However, although the exploration of the pathogenesis and treatment strategy of HS has been ongoing, the pathogenesis of HS has not been fully elucidated so far, and effective treatments are lacking.

According to the presence or absence of labor-related factors, the HS can be divided into classic heat stroke (CHS) and external heat stroke (EHS) [1]. CHS mainly occurs in the elderly, children, and people with underlying diseases [4], which is caused by the dysfunction of the thermoregulation in a high-temperature environment, while EHS mainly occurs in military officers, soldiers and young adults who perform high-intensity physical work in a high-temperature and high-humidity environment.

Clinically, HS is identified as a syndrome of extremely high fever, bleeding and coagulation disorders, circulatory failure, systemic inflammatory reaction, and multiple organ dysfunction [5]. In addition to the direct injury caused by heat exposure, the key pathophysiological changes induced by systemic inflammatory response syndrome (SIRS) [6] and multiple organ dysfunction syndrome (MODS) are also important pathological mechanisms of HS. Among the injured target organs, acute liver injury (ALI) and its more serious form acute liver failure (ALF) are well-documented complications of HS [7, 8] and served as a direct cause of HS patient death [9, 10]. A large number of hepatocytes undergo degeneration changes during the pathological process of HS [11].

The studies have shown that one of the important mechanisms of HS is the excessive opening of intestinal tight junctions, the destruction of intestinal cell structure and function, the increase in intestinal mucosal permeability, and the introduction of endotoxin into the blood [12, 13]. The blood from the gastrointestinal tract must pass through the portal vein system to the liver before entering the circulation, which enables the liver to play a vital role in metabolism, immunity, excretion, and other aspects [14]. Therefore, the liver not only plays a victim role in the course of HS, but also is a key factor in the pathogenesis of HS.

**Fig. 1:**
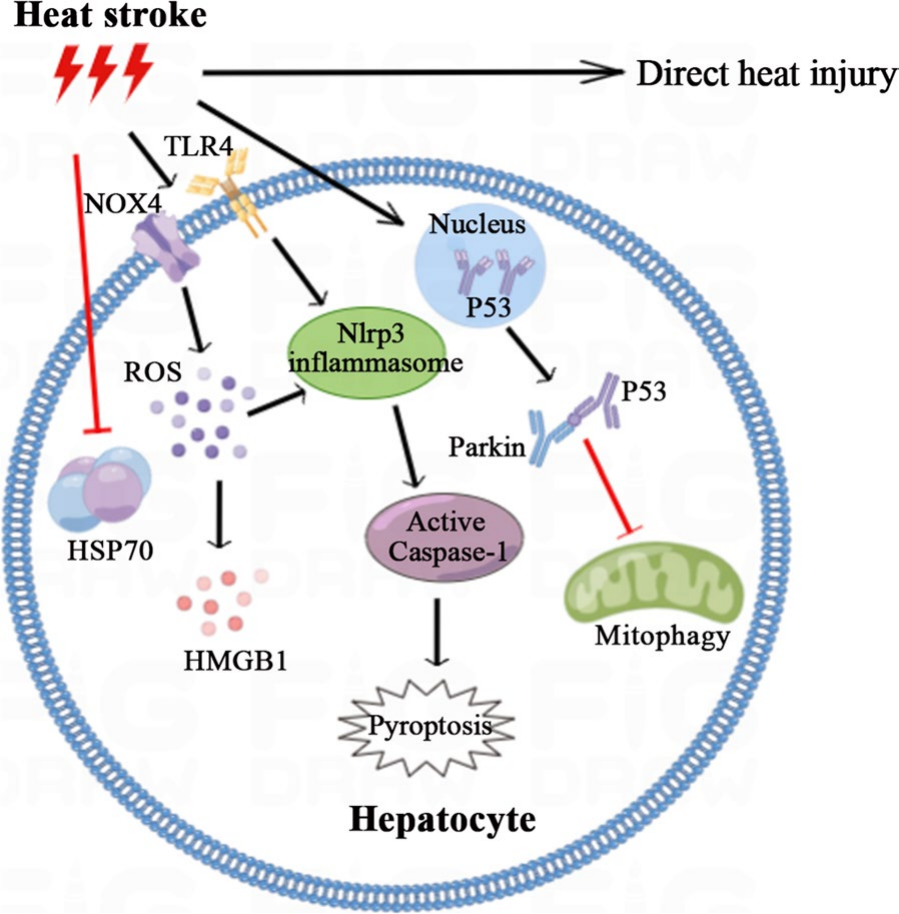
The related mechanism of hepatocyte injury during HS-induced liver injury. In addition to direct heat injury, HS can cause liver injury through various mechanisms, including inhibiting the expression of HSP70 of hepatocytes, promoting the production of ROS, promoting the inflammatory response and pyropsis, etc.

**Figure2:**
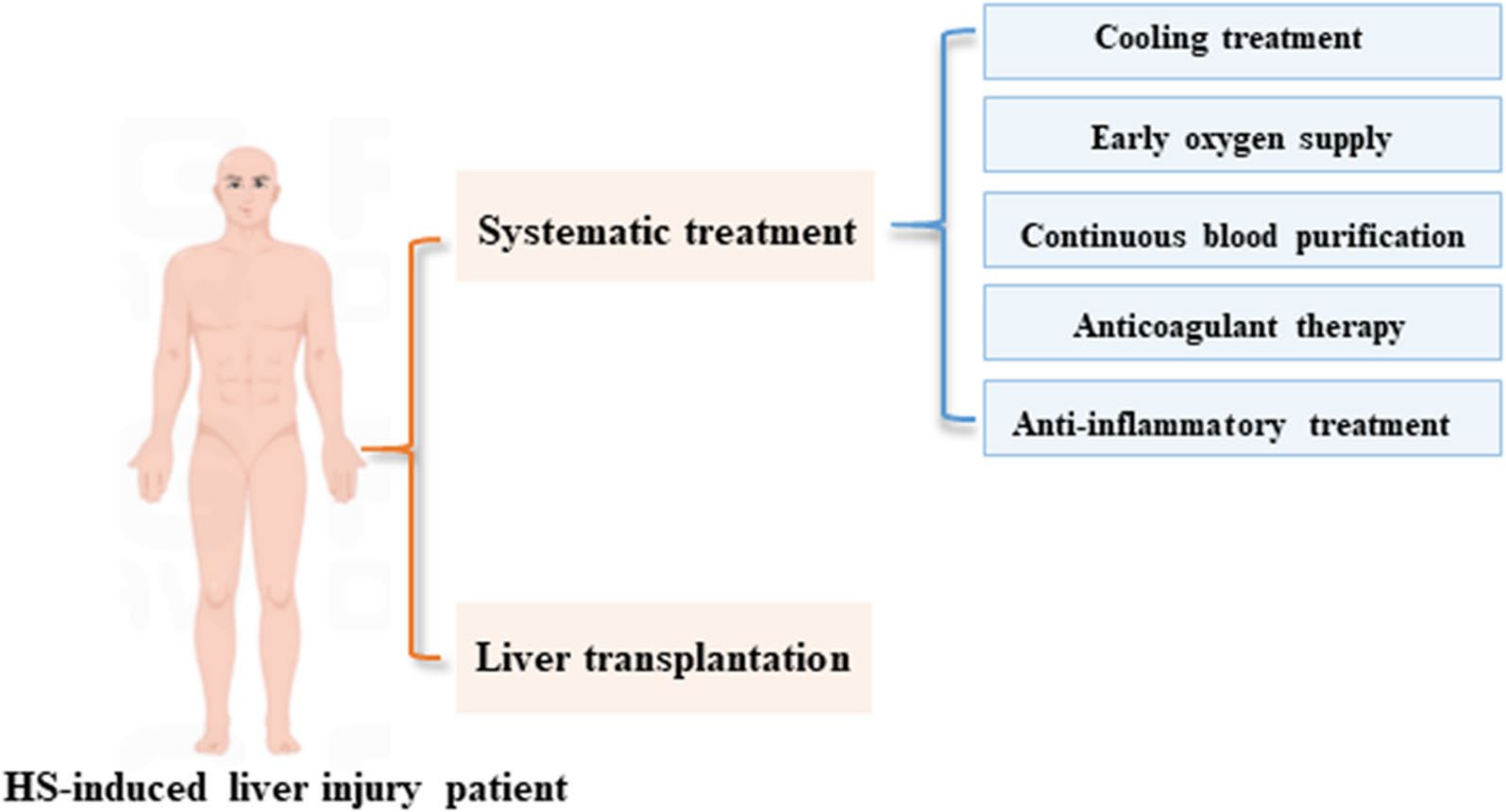
The main treatment strategies for HS-induced liver injury. It mainly focuses on systematic support treatment, including cooling treatment, early oxygen supply, CBP, anticoagulant therapy, and anti-inflammatory treatment. For those with irreversible liver injury, liver transplantation provides a possible choice

In this review, we systematically elaborated the possible mechanisms (as shown in Fig. 1) and treatment strategies (as shown in Fig. 2) of HS-induced liver injury, thus providing a theoretical basis and further research direction for the further study of HS-induced liver injury.

## The related mechanisms of HS-induced liver injury.

### The excessive inflammatory response

Excessive inflammation is an important cause of MODS in HS, while the translocation of endotoxin in the intestinal cavity caused by intestinal injury into the circulation is the key factor to trigger SIRS and MODS including liver injury in HS. Both animal and human studies have suggested that heat stress can cause serious damage to intestinal barrier function [15, 16]. On the one hand, heat stress can induce oxidative stress, nitrogen stress, and other negative cellular reactions in the body, and ultimately lead to intestinal epithelial cell damage and apoptosis [17, 18]. On the other hand, the maintenance of intestinal barrier function requires both active intestinal epithelial cells and the normal expression of intercellular tight junction proteins, while HS can lead to the reorganization of the intestinal cytoskeleton and downregulate the expression of tight junction proteins between intestinal cells [19, 20], thus leading to intestinal barrier dysfunction and the translocation of bacteria and endotoxin from the intestinal tract to the systemic circulation.

Cytokines are considered to be key mediators of SIRS in HS-induced systemic MODS [21] and are closely related to the severity and outcome of HS [22, 23]. Although many proinflammatory cytokines such as interleukin (IL)-1β, IL6, tumor necrosis factor-alpha(TNF-α), IL-8, etc., were found to be significantly increased in HS patients in the early years [23–25], the research on the pathogenesis of excessive inflammation in HS has not been elucidated.

As one of the most ubiquitous, abundant, and evolutionarily conserved transcription and growth factors in eukaryotes [26], high mobility group box 1 (HMGB1) occupies an important position in the diagnosis and treatment of HS. Consistent with multiple septic [27] or non-septic systemic inflammation [28], HMGB1 levels in HS patients were positively associated with disease severity and mortality [29]. Antithrombin III, thrombomodulin (TM), and hypothermia treatment could decrease the level of HMGB1, inhibit excessive inflammation, and improve HS-induced organ damage [30–32]. More precisely, Wang et al. used HMGB1 monoclonal antibody to specifically inhibit HMGB1 to explore the role of HMGB1 in HS-induced ALI [33]. They found that the intervention of the HMGB1 monoclonal antibody could significantly improve liver function and alleviate liver damage in the HS rat model. The mechanism of HMGB1 in the HS liver injury model was further explored by Yan Geng et al. [34]. As a typical damage-associated molecular patterns (DAMPs) molecule, HMGB1 can mediate HS-induced activation of the Nlrp3 inflammasome through the Toll-like receptor (TLR) 4 and receptor for advanced glycation end products (RAGE) signaling, thereby inducing the activation of IL-1β, as well as hepatocyte pyroptosis and consequent severe liver injury, while the HMGB1 inhibition, silencing of Nlrp3, or blockade of caspase-1 could significantly prevent the Nlrp3 inflammasome activation, thereby reducing liver injury in HS-induced liver injury.

Macrophages and monocytes activated by proinflammatory factors are important sources of HMGB1 [35], while HMGB1 can also leak from necrotic or damaged cells [36, 37]. Under heat stress, HMGB1 expression in liver macrophages and plasma increased significantly. The TM intervention could decrease the plasma HMGB1 levels even with delayed treatment [38]. These findings indicated that TM in HS can not only improve HS-induced liver injury by improving the systemic coagulation state but also effectively inhibit the excessive inflammatory reaction.

### Coagulation disorders

The activation of coagulation and fibrin deposition secondary to inflammation can be regarded as an important part of the host's defense against high fever, but the intensification of systemic inflammatory reaction may lead to systemic blood coagulation activation and microvascular failure, thus exacerbating organ dysfunction [39]. HS can cause obvious coagulant status disorder even more obvious than sepsis. HS-activated coagulation was indicated by the prothrombin time, activated partial thromboplastin time, and D-dimer levels, and decreased platelet count [40]. In HS patients, the whole-blood tissue factor, TAT, and soluble TM levels dramatically increased, while the levels of the major physiologic anticoagulants were significantly decreased, including antithrombin, protein C, and protein S [39]. TM can bind thrombin to convert protein C to activated protein C(APC) [41, 42].

TM is an endothelial anticoagulant cofactor, which exerts an important role in the regulation of intravascular coagulation [43]. In addition to regulating the blood coagulation state of the whole body, the complex formed by TM and thrombin can promote the activation of protein C, which has the effect of inhibiting monocytes and macrophages [44, 45]. The positive role of APC in HS in reducing systemic inflammation, hypercoagulability, and organ damage has been confirmed [46]. To sum up, TM can effectively improve liver injury in HS, which may involve many mechanisms. TM supplementation should be considered an important treatment strategy for patients with HS.

Endothelial cells play an important role in maintaining vascular homeostasis and normal physiological function [47]. Endothelial cell injury is one of the most important pathological features of HS. Intense heat stress could induce obvious endothelial cell damage and apoptosis [48, 49]. In addition to causing the release of cytokines, which further amplifies the inflammatory response [50], the damaged endothelial cells will lead to the exposure of collagen fibers under the endothelium, activate coagulation factor XII, and then activate fibrinogen to form fibrin thrombus, leading to endogenous coagulation dysfunction [51]. At the same time, endothelial cells can release tissue factors and activate coagulation factor VII, leading to exogenous coagulation dysfunction. Furthermore, studies showed that the reduction of endothelial cell apoptosis was closely related to the improvement of coagulation disturbances in HS, which can effectively reduce HS-induced liver injury [52, 53]. These findings suggested that endothelial cells could be an important target for improving HS-induced liver injury.

### Abnormal hepatocyte death

The most common pathological change of HS-induced liver injury is the massive degeneration of hepatocytes including abnormal cell death [11], while pyroptosis exerts an important role in abnormal cell death in HS-induced liver injury [34]. Pyroptosis is a caspase-1-dependent programmed cell death characterized by cell swelling, rapid plasma membrane rupture, and release of proinflammatory intracellular substances [54, 55]. NOD-like receptor family pyrin domain containing3 (NLRP3) is an intracellular pattern recognition receptor, which was involved in cell pyroptosis by assembling into an inflammasome [56]. NLRP3-dependent pyroptosis is an important cause of abnormal cell death in HS-induced liver injury [34]. HS-induced liver injury is accompanied by the activated inflammasome, which can effectively induce the activation of IL-1 β and hepatocyte pyroptosis, thus leading to severe liver injury.

Excessive ROS will be produced during heat stress [57], which has been proven to be a key stimulator of NLRP3 inflammasome and a potential target for negative regulation of cell pyroptosis [58]. Ming Zhang et al. further found that the overproduction of ROS and NLRP3-dependent pyroptosis is an important pathological mechanism in HS-induced liver injury [59].

The angiotensin peptides are the important components of the renin-angiotensin system (RAS). The previous studies have confirmed the value of Ang II and Ang—[1–7] as potential biomarkers in inflammatory diseases [60, 61]. The level of angiotensin peptides changed during HS and appears to be associated with excessive ROS production [62]. In the HS-induced liver injury, the expression of Ang II increased, while the levels of Ang—[1–7] decreased, which were consistent with their receptors and converting enzymes [59]. As an analog of Ang- [1–7], AVE 0991 can significantly inhibit the production of ROS and reduce the protein levels of NOX4, NLRP3, caspase-1, and IL-1 in HS-induced liver injury. These findings indicated that RAS exerted an important role in HS-induced liver injury, while the mechanism is closely related to the excessive production of ROS and the consequent induction of cell pyroptosis.

Another mechanism of cell death in HS is the Z-DNA binding protein 1 (ZBP1)-mediated programmed cell death. As a Z-nuclear acid sensor, ZBP1 can activate receptor-interacting protein kinase 3(RIPK3)-mixed lineage kinase domain-like(MLKL) pathway-dependent programmed cell necrosis [63, 64]. Recently, the research of Fangfang Yuan et al. suggested that the expression of ZBP1 in HS was significantly increased and could induce RIPK-dependent cell death. The deletion of ZBP1 and RIPK3 can significantly reduce cell apoptosis in HS and effectively alleviate multiple organ damage including liver injury. Further, Fangfang Yuan et al. found that heat stress can enhance the activation and occupation of heat shock transcription factor1 (HSF1) binding sites in the ZBP1 promoter. Consistently, the deletion of HSF1 can inhibit the increase in ZBP1 expression and cell death induced by heat stress.

Mitophagy plays a crucial role in regulating homeostasis in the liver [65, 66]. As the most important mechanism for self-regulating mitochondrial quality control and selectively clearing the damaged mitochondria [67], the dysfunction of the mitophagy leads to the accumulation of the dysfunctional mitochondria, the excessive activation of apoptosis-inducing factors, and the excessive release of the ROS, thus leading to abnormal cell apoptosis [68]. The previous studies have suggested that mitophagy is closely related to the occurrence and development of various liver diseases, including viral hepatitis, liver ischemia/reperfusion (I/R) injury, and drug-induced liver injury [69]. In HS-induced liver injury, the important role of mitophagy has also been confirmed. P53 has been well characterized for its response to regulate the cell cycle and cell apoptosis [70, 71]. Under the stimulation of heat stress, the translocation of P53 from the nucleus to the cytoplasm is significantly increased, thereby binding to Parkin to reduce Parkin’s translocation from the cytosol to the mitochondria, which decreases mitophagy activation and exacerbates apoptosis in hepatocytes, while the inhibition of p53 with siRNA or PFT-a significantly suppressed the aberrant apoptosis in HS-induced ALI [72].

### The abnormal production and release of extracellular vesicles of hepatocytes.

Extracellular vesicles (EVS) are novel mediators of intercellular communication [73]. Various biologically active substances, including proteins, lipids, and nucleic acids, can be transferred to recipient cells via EVS and modulate their biological processes and function [74]. EVS is a key event in liver pathology [74] and function in a variety of liver diseases, regulating physiological cellular events [75–77].

In HS-induced ALI, HS could lead to a marked increase in EVS released from hepatocytes, which promotes liver injury both in vitro and in vivo, while the EVS synthesis inhibitor GW4968 attenuated HS-induced liver injury [78]. Furthermore, the proteomic analysis suggested that the regulation of programmed cell death was the most distinctly altered pathway [78]. Necroptosis is another form of programmed cell death that was controlled by receptor-interacting protein 1 (RIP1), RIP3, and MLKL and was caspase-independent [79]. Not the same as apoptosis, necroptosis is accompanied by an increase in plasma membrane permeability, which can lead to the release of DAMPs, such as HMGB1 and mitochondrial DNA [80], that trigger powerful immune and inflammatory responses [81, 82]. Both in vitro and in vivo experiments confirmed that EVS of hepatocytes in HS can promote necroptosis and apoptosis.

Exosomes are EVS that mediate the transport of a variety of bioactive molecules that regulate the function of target cells [83, 84]. Hepatocytes are the main source of exocrine bodies in the liver. Studies have demonstrated that hepatocytes are not only victims of harmful stimuli, but also actively participate in liver injury by releasing “danger signals” [85].

## The dysregulation of Kupffer cells

Liver-resident macrophages/Kupffer cells (KCs) are central to maintaining liver homeostasis [86]. In response to heat stress in the liver, KCs exert a key role in the clearance of gut-derived endotoxin by phagocytosis, while the dysfunction of KCs is the main explanation for the elevated endotoxin concentration [87]. However, at the same time, KCs are also the main source of inflammatory factors in the liver [88]. TNF-α, IL-β, and IL-6 secreted by KCs were significantly increased in HS, and inhibiting the secretion of KCs appeal-related factors can effectively alleviate HS-induced liver injury [89].

Macrophage inflammatory protein-1α (MIP-1α) is an important chemokine in the inflammatory response, which can effectively activate immune cells, and regulate the synthesis of cytokines [90]. The various immune cells, including neutrophils, macrophages, and lymphocytes, are important sources of the production of MIP-1α [91]. Accounting for 80–90% of the total resident macrophages in vivo, KCs are the main source of the MIP-1α in the liver [92]. The previous studies have suggested that MIP-1α secreted by KCs was related to the excessive inflammation that leads to MODS in ischemia–reperfusion injury. Further evidence showed that MIP-1α can promote peritoneal macrophages to secrete tumor TNF-α, IL-1β, and IL-6 [93]. Wang et al. found that there were elevated MIP-1α expression and TNF-α, IL-1β, and IL-6 in the KCs in the HS-induced liver injury, while the activation of JNK signaling is required for this pathological process. JNK plays an important role in the inflammation response, cell apoptosis, and heat stress, especially in the KCs [94, 95]. In vitro and in vivo, after inhibiting JNK phosphorylation in KCs with sp600125, the inflammatory cytokines and MIP-1 produced by KCs were significantly reduced [96]. Interestingly, the inhibition of JNK phosphorylation level and MIP-1α was accompanied by the increase in KCs phagocytosis.

### The dysregulation of heat shock protein.

Different defense systems protect cells from heat injury, the most important of which is the increased expression of HSP [97, 98]. The increased expression of HSP level is related to the better prognosis of HS patients [99]. HSP is a family of highly conserved proteins that function as molecular chaperones by promoting protein folding and refolding, mediating transmembrane transport of some secreted proteins, and targeting proteins for lysosomal degradation [97]. Among them, the expression of HSP70 can effectively prevent organ damage [100]. The pre-treatment of 17‐dimethylaminoethylamino‐17‐demethoxy‐geldanamycin(17‐DMAG) could upregulate the expression of HSP70 in the liver, thereby significantly alleviating the HS-induced liver dysfunction in rats [101]. Furthermore, the symptoms of hypotension and tachycardia could also be markedly improved.

As is a highly conserved nuclear zinc-finger DNA-binding protein, the enzyme poly(ADP-ribose) polymerase-1 (PARP-1) has long been considered the key factor of DNA damage sensor and DNA repair system [102]. In recent years, it has been shown that genetic deletion or pharmacologic blockade of PARP-1 could significantly inhibit the excessive inflammation response [103–105]. Furthermore, the inhibition of PARP can reduce various forms of liver injury [106, 107]. In HS-induced liver injury, PARP blockage has also emerged as a promising treatment [108]. The level of IL-1β and IL-6 expression in PARP−/− mice and mice treated with PARP inhibitors was significantly lower than that in control mice after heat exposure. The PARP inhibition could significantly increase the expression of HSP70 and HSP27 at messenger RNA and protein levels. Although the upregulation of HSP protein and the inhibition of PARP showed consistency in HS-induced liver injury, the exact molecular mechanism remains unclear. José Yélamos et al. proposed two possible explanations. One possibility is that PARP-1, as a part of the complex of histone variant mH2A1.1, is related to the hsp70.1 promoter, which can inactivate the expression of hsp70.1 [109]. Another possibility is that PARP-1 destroys the DNA binding of heat shot factor-1 and the promoter of HSP genes, which has been confirmed in fibroblasts [110].

## The treatment strategy for HS-induced liver

Although proper hypothermia and active treatment have certain effects, the liver injury still occurs frequently in HS patients and acts as a direct cause of death [10]. As a life-threatening condition, patients with HS could be fatal if appropriate evaluation and treatment are not initiated promptly. Many active intervention strategies have been proven to have positive effects on improving the symptoms and prognosis of HS patients. Here, we focus more on the published research on the treatment of HS-induced liver injury to make a more in-depth and systematic exposition.

### Systematic treatment

As a systemic disease, the treatment of HS currently relies more on supportive treatment [111]. Medical therapy and supportive treatment are the first-line treatment strategies for HS. We summarized the main and generally accepted treatment strategies for HS at present, as follows:

***Cooling*** Early and rapid cooling is of great significance to the prevention of irreversible tissue damage and improves the prognosis of HS patients [112, 113]. Various cooling strategies including the alcohol bath, hibernation mixture, bedside blood filtration, and cold liquid intravenous drip can rapidly reduce the patient's temperature. There is no evidence to support the superiority of any cooling technology in HS. The effects of non-invasive, evaporative, or conductive-based cooling techniques, whether alone or in combination, seem comparable [112].

*Early oxygen supply* Cerebral ischemia and hypoxia, and nervous system dysfunction are important characteristics of patients with HS [114]. Hyperbaric oxygen therapy (HBOT) is an effective method to treat brain injury. HBOT can improve circulation, significantly improve tissue ischemia and hypoxia, and reduce brain edema. In addition, HBOT can also exert other positive molecular biological effects, such as anti-inflammatory and antioxidant stress, and inhibit apoptosis [115]. So when the patient's vital signs are stable, hyperbaric oxygen treatment can be carried out as soon as possible.

*Continuous blood purification (CBP)* Simple plasma exchange (PE), continuous hemodialysis filtration (CHDF), and their combination are commonly used to treat severe HS with MODS [116–118]. With the help of cardiopulmonary bypass technology, CBP can achieve an ideal cooling effect compared with traditional physical cooling. It can not only reduce the catabolism of the body but also effectively eliminate the inflammatory mediators such that IL-1 and IL-2 can maintain the homeostasis of the body, which is helpful for the recovery of HS patients. CBP can significantly improve the prognosis of patients with HS, improve the survival rate of patients, and is an important means of treating HS [119].

*Anticoagulant therapy* In the early stage of the onset of HS patients, high fever leads to the loss of a large number of body fluids, resulting in blood concentration. At the same time, hyperthermia causes microvascular endothelial cell damage, leading to the activation of the endogenous coagulation pathway. Subsequently, fibrin is deposited in arterioles and capillaries, which, together with platelet aggregation, leads to intravascular micro thrombosis.

*Anti-inflammatory treatment* Systemic inflammation and coagulopathy are the two main factors that cause life-threatening organ dysfunction during HS [120]. The pathophysiological mechanism of HS is similar to sepsis, and systemic inflammatory reaction is important pathogenesis. Many drugs that have been proven to have definite anti-inflammatory effects, such as dexmedetomidine and melatonin, have positive effects on improving multiple organ damage and the prognosis of HS [121–123].

### Liver transplantation

Although the mild or moderate liver injury is the most common in HS patients [124], only a few patients have serious liver injury that may lead to fatal consequences [8]. In these cases, the drug treatment and other active supportive treatments are ineffective, and the liver injury caused by HS progresses to ALF with organ dysfunction. At this time, liver transplantation can provide a treatment option.

A clinical observational study showed that in patients with HS-induced liver injury requiring mechanical intubation and continuous renal replacement therapy, liver transplantation can immediately restore renal function [125], In the follow-up several years after the operation, the liver function was normal without any complications after transplantation.

According to Philippe Ichai et al., the classical standard of liver transplantation does not seem to apply to HS-induced ALF [113]. And they emphasized that the decision to implement LT should not be made hastily. Even in the case of severe liver failure, the fluctuation of prothrombin time and the clinical condition of patients should also be considered. They believed that PT lasted < 10% after the onset of heat stroke, and there was no sign of elevation after 3 days of median time, which was an important reference factor for LT implementation.

## Discussion

HS is a syndrome with complex pathological mechanisms including microvascular injury, thrombosis, inflammation, and cell apoptosis [126]. Among the MODS caused by HS, the liver is considered to be one of the first organs to be damaged [127]. Liver damage occurs in nearly all cases of HS and is often the site of fatal damage [128]. Although a large number of clinical and basic medical researches on HS have been carried out in recent years, most of the underlying pathological mechanisms have not been precisely understood due to the complexity of HS. In the current clinical practice of HS treatment, more systemic treatments such as plasma exchange and hypothermia are used, but specific treatments for HS-induced ALI are lacking. The liver, as the key line of defense for the collective elimination of endotoxin, may obtain surprises with specific treatment for HS-induced ALI.

At present, the research on HS is mainly limited by two aspects. One is the complex pathological mechanism of HS. Secondly, although the incidence rate of HS is increasing year by year [129], the number of patients with HS, especially those with severe multiple organ dysfunction and even death, is still relatively less. Therefore, at present, there is no large cohort of clinical studies to provide accurate evidence-based medical evidence for the pathogenesis and treatment of HS. For example, although it is generally recognized that early and rapid cooling treatment is of positive significance for patients with HS. However, so far, no evidence has been found for a specific end-point temperature at which cooling can be safely stopped. The previous studies have shown that the conduction-based cooling method, that is, immersion in ice water has a positive effect on labor heat stroke, and Wang et al. proposed the use of 38.6° C as a safe rectal temperature cooling limit for the cold water bath, Wang et al.'s research is based on the subjects whose core temperature reaches 39.5 through temperature and exercise, not HS patients [130].

In brief, the mechanism of HS-induced liver injury is not only related to systemic factors such as systemic inflammatory reaction and coagulation dysfunction but also closely related to pathological mechanisms such as abnormal death of liver cells and abnormal function of KCs. Therefore, for the treatment of HS-induced liver injury, we should not only implement systematic and supportive treatment but also target the liver for precise treatment. We need to conduct more and more accurate research to reveal the mechanism of HS-induced and find effective treatment strategies.

## Future perspective

In the past decades, a large number of studies on the pathogenesis and treatment strategies of HS have indeed revealed many possible pathogenesis and treatment targets; however, most of the studies are at the animal level. Besides liver transplantation, there are few liver-targeted therapies for HS patients. Therefore, in our view, the future focus of research on HS-induced liver injury is the clinical verification of relevant research, especially the data integration of liver-targeted therapy and its efficacy. Further studies, including clinical randomized controlled trials, are expected.

## Data Availability

Not applicable.

## Abbreviations

ALI: Acute liver injury
ALF: Acute liver failure
APC: Activated protein C
CHS: Classic heat stroke
CHDF: Continuous hemodialysis filtration
DAMPs: Damage-associated molecular patterns
EVS: Extracellular vesicles
EHS: External heat stroke
HSP: Heat shock protein
HSF1: Heat shock transcription factor 1
HS: Heat stroke
HMGB1: High mobility group box 1
HBOT: Hyperbaric oxygen therapy
I/R: Ischemia/reperfusion
IL: Interleukin
KCs: Kupffer cells
MIP-1α: Macrophage inflammatory protein-1α
MLKL: Mixed lineage kinase domain-like
MODS: Multiple organ system failure
NLRP3: NOD-like receptor family pyrin domain containing3
PARP-1: Poly(ADP-ribose) polymerase-1
RAGE: Receptor for advanced glycation end products
RIP1: Receptor-interacting protein 1
RIPK3: Receptor-interacting protein kinase 3
RAS: Renin-angiotensin system
PE: Simple plasma exchange
SIRS: Systemic inflammatory response syndrome
TM: Thrombomodulin
TLR: Toll-like receptor
TNF-α: Tumor necrosis factor
ZBP1: Z-DNA binding protein 1
17‐DMAG: 17‐Dimethylaminoethylamino‐17‐demethoxy‐geldanamycin
